# Migraine attacks the Basal Ganglia

**DOI:** 10.1186/1744-8069-7-71

**Published:** 2011-09-21

**Authors:** Nasim Maleki, Lino Becerra, Lauren Nutile, Gautam Pendse, Jennifer Brawn, Marcelo Bigal, Rami Burstein, David Borsook

**Affiliations:** 1 Department of Radiology, Children's Hospital Boston, Harvard Medical School, Boston, MA, 02115, USA; 2 Department of Psychiatry, P.A.I.N. Group, Brain Imaging Center, McLean Hospital, Harvard Medical School, Belmont, MA, 02478, USA; 3 Departments of Psychiatry and Radiology, Massachusetts General Hospital, Harvard Medical School, Charlestown, MA, 02129, USA; 4 Merck Investigator Studies Program and Scientific Education Group, Merck Research Laboratories, Merck & Co., Inc., North Wales, PA, 19454, USA; 5 Department of Neurology, Albert Einstein College of Medicine, Bronx, NY, 10461, USA; 6 Department of Anesthesia and Critical Care, Beth Israel Deaconess Medical Center, Harvard Medical School, Boston, MA, 02115, USA

**Keywords:** Headache, Pain, Migraine, fMRI, Functional Connectivity, Morphometry, Gray Matter Volume, Basal Ganglia

## Abstract

**Background:**

With time, episodes of migraine headache afflict patients with increased frequency, longer duration and more intense pain. While episodic migraine may be defined as 1-14 attacks per month, there are no clear-cut phases defined, and those patients with low frequency may progress to high frequency episodic migraine and the latter may progress into chronic daily headache (> 15 attacks per month). The pathophysiology of this progression is completely unknown. Attempting to unravel this phenomenon, we used high field (human) brain imaging to compare functional responses, functional connectivity and brain morphology in patients whose migraine episodes did not progress (LF) to a matched (gender, age, age of onset and type of medication) group of patients whose migraine episodes progressed (HF).

**Results:**

In comparison to LF patients, responses to pain in HF patients were significantly lower in the caudate, putamen and pallidum. Paradoxically, associated with these lower responses in HF patients, gray matter volume of the right and left caudate nuclei were significantly larger than in the LF patients. Functional connectivity analysis revealed additional differences between the two groups in regard to response to pain.

**Conclusions:**

Supported by current understanding of basal ganglia role in pain processing, the findings suggest a significant role of the basal ganglia in the pathophysiology of the episodic migraine.

## 1. Background

Migraine is a common neurological disorder, frequently starting in childhood and extending into adulthood. It is defined by recurrent headaches that last 4-72 hours and affect patients one to fourteen times each month in the episodic form and more than fourteen attacks per month in the chronic form. Most patients seeking medical help are not responsive to current preventive therapies [1] that could mitigate such progression. To identify neurological reasons for migraine disease, we attempted to compare brain functions and morphology in patients at the two ends of episodic migraine spectrum: those with very low frequency of migraine attacks vs. those with very high frequency of migraine attacks.

Numerous imaging studies of migraine patients have described multiple changes in brain functions as a result of migraine attacks: these included enhanced cortical excitability [2], increased gray matter volume in some regions and decreased in others, [3,4]; enhanced brain blood flow [5-7]; and altered pain modulatory systems [8-10].

The Basal Ganglia (BG) are a major site for adaptive plasticity in the brain, affecting in the normal state a broad range of behaviors [11] and neurological and psychiatric conditions [12] including pain [13,14]. The BG seem to be involved in the integration of information between cortical and thalamic regions and in particular the three domains of pain processing - sensory, emotional/cognitive and endogenous/modulatory. More recent evidence points to BG being involved through direct connections from sensory inputs (including pain (see Borsook et al., 2010) and not involving cortical loops [15]. The BG may have a role in that they may be involved in habit and stimulus-response learning [16]. Such learning may be derived from pain related regions involved in sensory (e.g., S1), affective (e.g., cingulate or anterior insula) or cognitive regions (e.g., medial and lateral prefrontal cortices).

Brain imaging studies of migraineurs have shown decreased activation in the BG of migraineurs vs. controls [17], increased activation (blood flow) in the BG during the ictal state and lesions in the BG of migraineurs [18,19]. This is the first study in which attempt is made to compare brain functions of non-progressing patients with those of progressing patients. In comparing the two groups of patients such alterations may provide opportunities to predict which patients progress.

## 2. Results

### 2.1. Demographics

Demographic characteristics for each cohort are noted in Table 1. Low and high frequency migraine patients (N = 10 each, 3 male and 7 female) were matched for gender, and age and there were no significant differences between the age (HF: 43.2 ± 3.4 (mean ± SD), LF: 40.2 ± 3.6 (mean ± SD), (p = 0.46)) or age of onset (HF: 24.2 ± 4.4 (mean ± SD), LF: 21.6 ± 3.2 (mean ± SD), p = 0.59)) between the two cohorts. One patient in each cohort had migraine with aura and another patient in the LF cohort experienced auras occasionally with the migraine attacks. The HF cohort on average had used at least 5.6 greater triptan use during the course of their migraine disease. There was a significant difference between the average numbers of migraine attacks experienced in LF (1.7 ± 0.5 (mean ± SD) attacks per month) vs. HF (9.3 ± 2.6 (mean ± SD) attacks per month) migraqineurs (p < 0.0001).

**Table 1:** Demographic Data of the Studied Subjects

Group	Age	Age of Onset	BDI Score	Abortive Rx	Analgesic Rx	Preventive Rx
**High**	43.9 ± 3.37	24.2 ± 4.4	1.9 ± 2.4	80%	80%	20%

**Low**	40.2 ± 3.60	21.6 ± 3.2	2.1 ± 2.5	80%	50%	10%

Abortive Rx includes: Rizatriptan, Sumatriptan, and Naratriptan; Analgesic Rx includes: Iboprufen, Excedrine migraine, and Advil, and Preventive Rx includes: Amitrityline and Verapamil.

### 2.2. Psychophysical/Biometric Data

**Figure 1:**
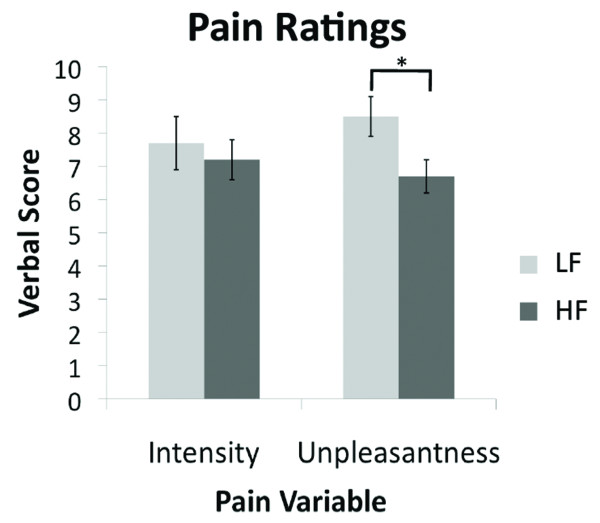
**Migraine Pain Intensity and Unpleasantness Ratings**. There is a significant difference in the pain unpleasantness scores between the two cohorts (p < 0.028). The scores are based on a 0-10 subjective scale for migraine pain intensity and pain unpleasantness.

There were no significant differences in migraine headache intensity (LF: 7.7 ± 2.4 (mean ± SD), HF: 7.2 ± 1.8 (mean ± SD), (p = 0.61)). The migraine headache unpleasantness rating however was significantly different between the two groups (LF: 8.5 ± 1.8 (mean ± SD), HF: 6.7 ± 1.4 (mean ± SD), (p < 0.028)), Figure 1. The average QST pain thresholds were not significantly different (LF: 46.06 ± 4.26°C (mean ± SD), HF: 45.89 ± 2.77°C (mean ± SD), (p = 0.83)). The average VAS scores for individually tailored threshold + 1°C temperatures applied to the dorsum of the hand were not significantly different for pain intensity (LF: 8.1 ± 2.25 (mean ± SD), HF: 6.17 ± 3.4 (mean ± SD), (p = 0.27)) or for pain unpleasantness (LF: 6.32 ± 2.58 (mean ± SD), HF: 5.47 ± 3.2 (mean ± SD), (p = 0.64)), although there was a trend.

### 2.3. MRI Measures

#### 2.3.1. Functional Analysis - Painful Heat fMRI Activation

**Figure 2:**
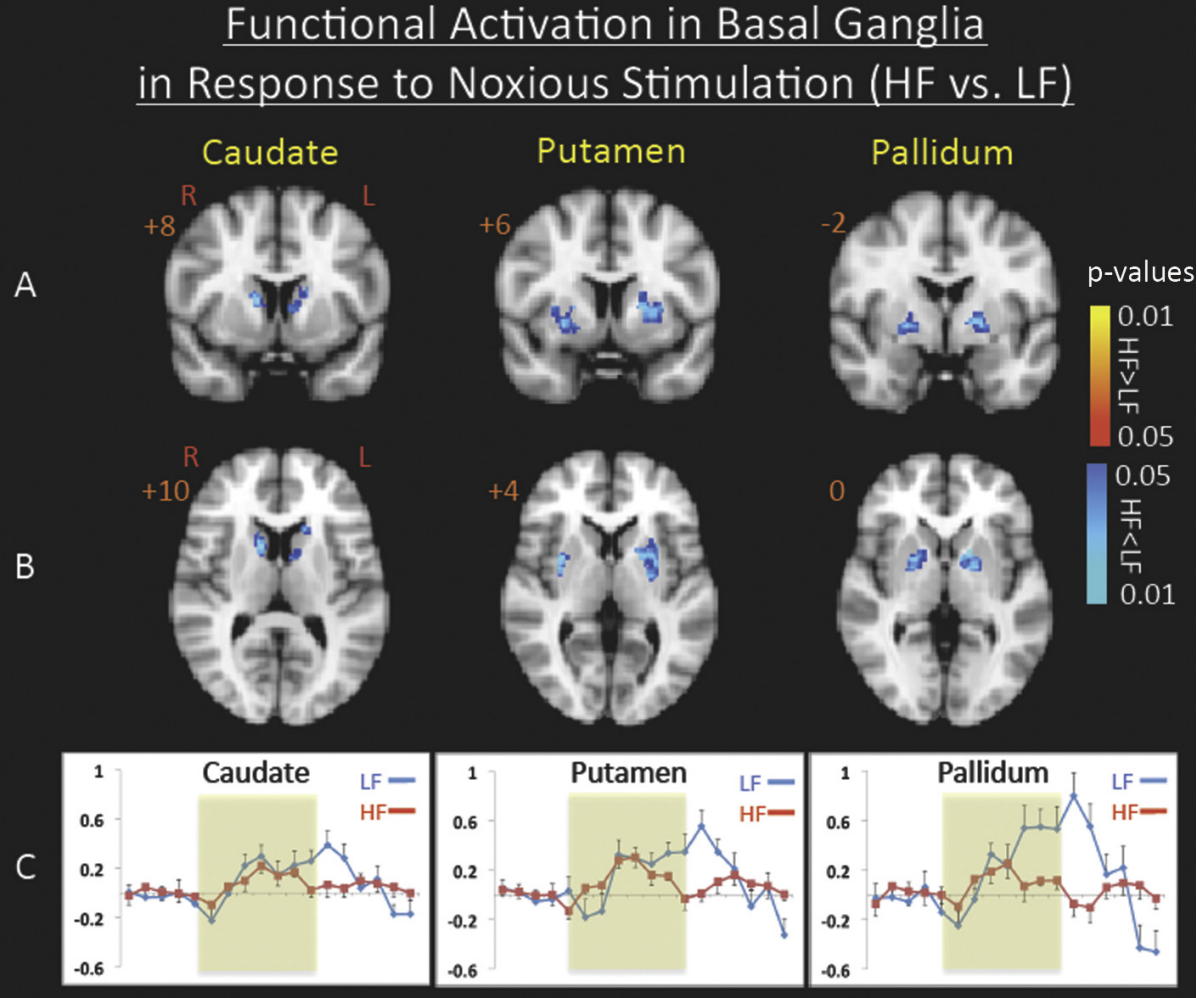
**Contrast Maps for Painful Heat fMRI Activation**. Contrast analysis of the HF vs. LF migraine group in response to the "pain threshold +1°C" stimuli revealed significant (p < 0.05, corrected) differences in the basal ganglia nuclei in coronal (A) and axial (B) views. In (C) Single trial averages for the response to painful heat stimulation in caudate, putamen, and pallidum are presented. Yellow represents the stimulus application period.

Although data for the entire brain were acquired, striking differences was observed in basal ganglia (BG) structure and function as described below:

Contrast analysis of the HF vs. LF migraine group in response to the "pain threshold +1°C" stimuli revealed significant (p < 0.05, corrected) lower (HF < LF) BOLD signal changes throughout the caudate, putamen, and pallidum nuclei of the BG (Figure 2, and Table 2) in HF vs. LF patients that were also symmetrical. Increased (HF > LF) BOLD signal change (p < 0.05, corrected) was observed in the contralateral nucleus accumbens (NAc). Single trial averages also revealed significant reduction in the second peak of the biphasic hemodynamic response of the caudate (p < 0.024), putamen (p < 0.002) and pallidum (p < 0.003) in response to noxious stimulation in the HF group relative to the LF group.

**Table 2:** Sub-cortical Clusters for Painful Heat fMRI Activation

Brain Region	**Lat**.	Max z-stat	X(mm)	Y(mm)	Z(mm)	Vol (cm^3^)
*HF > LF*						

Thalamus	L	2.2586	-10	-28	8	0.352

PAG		2.1464	-6	-32	-4	1.176

Pons		2.2187	0	-26	-36	0.232

Hypothalamus	L	2.8448	-10	-6	-12	1.568

*HF < LF*						

Caudate	L	-1.7572	-16	20	10	1.504

Caudate	R	-1.7704	18	20	0	2.024

Putamen	L	-1.9119	-24	4	4	1.2

Putamen	R	-2.1153	30	2	4	1.312

Pallidium	L	-2.1256	-20	-2	0	0.328

Pallidium	R	-1.7506	16	4	-2	0.39

#### 2.3.2. Structural Analysis

**Figure 3:**
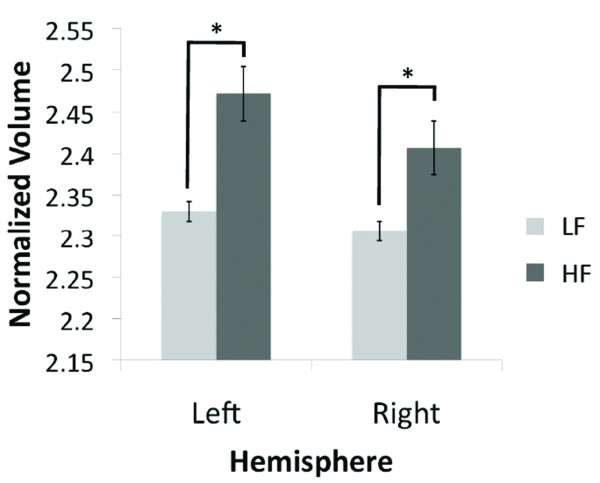
**Volumetric Changes of the Caudate**. The plots show the significant caudate volumetric differences in the high vs. low frequency migraine subjects (left: p < 0.025 and right: p < 0.006). The volumes have been normalized to the total intracranial volume to scale for the brain volume for each subject. Bar heights represent the mean value for each volumetric measurement. Error bars represent the 95% confidence interval of the mean. * denotes significance.

High frequency migraine patients showed a larger volume in the bilateral caudate vs. the low frequency migraine patients (left: p < 0.025 and right: p < 0.006), Figure 3. No other significant changes in subcortical structures were observed.

#### 2.3.3. Functional Connectivity (Fc) Analysis

**Figure 4:**
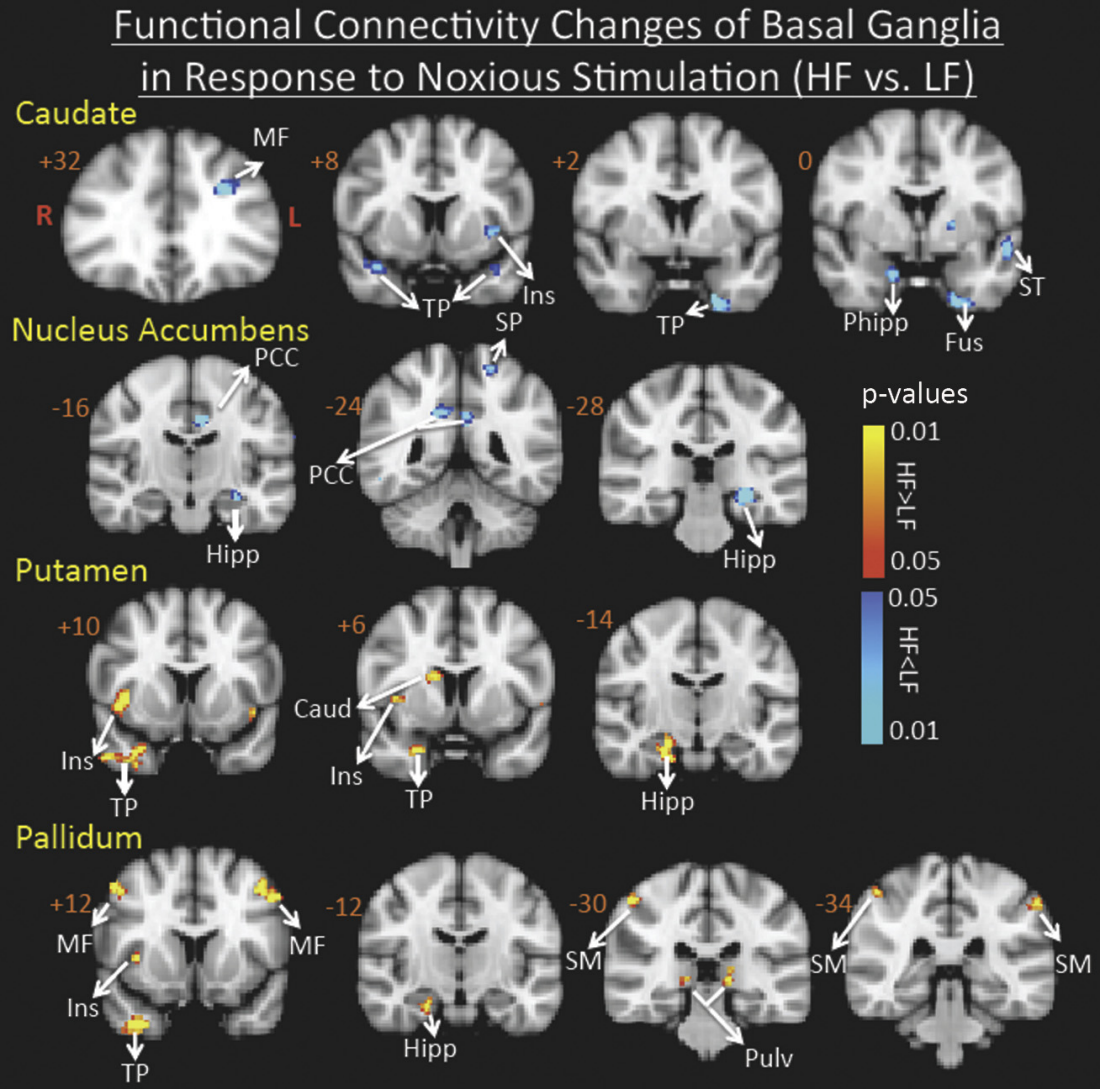
**Functional Connectivity Contrast Maps of the Basal Ganglia Nuclei**. Functional connectivity contrast maps of the basal ganglia nuclei during intermittent heat stimuli (pain threshold +1°C on hand) in high frequency migraine patients vs. low frequency migraine patients. *PCC: Posterior Cingulate Cortex, SM: SupraMarginal, SF: Superior Frontal, ST: Superior Temporal, SP: Superior Parietal, Ins: Insula, Hipp: Hippocampus, PHipp: Parahippocampus, Fus: Fusiform, Thal: Thalamus, Pulv: Pulvinar, TP: Temporal Pole, MF: Middle Frontal*.

The Fc analysis results, summarized in Figure 4 showed significant differences between the two groups encompassing a number of brain regions, further suggestive of widespread differences in brain function between the two groups as described in the following:

##### Caudate

Significantly reduced (HF < LF) Fc (p < 0.05, corrected) of caudate was observed with ipsilateral middle frontal gyrus, ipsilateral insula, bilateral temporal pole, and contralateral parahippocamus.

##### Putamen

Enhanced (HF > LF) Fc (p < 0.05, corrected) with putamen was observed in contralateral hippocampus, contralateral caudate, contralateral middle frontal gyrus and bilateral anterior insula (with a stronger connectivity contralaterally).

##### Globus Pallidus

Significantly increased (HF > LF) Fc (p < 0.05, corrected) with the pallidum was observed in bilateral middle temporal gyrus, bilateral supramarginal gyrus, bilateral thalamus, contralateral hippocampus, contralateral insula, and contralateral temporal pole.

##### Nucleus Accumbens (NAc)

Significantly reduced functional connectivity with NAc was observed in bilateral posterior cingulate cortex, ipsilateral superior parietal, and ipsilateral hippocampus.

## 3. Discussion

In this multimodal imaging study, in matched groups of HF and LF migraineurs, significant differences in gray matter volume and function in response to pain, as measured in the interictal period, were observed in the basal ganglia (BG). These regions are well positioned to integrate sensory, motor, cognitive and other information including behavior relating to predicting events, and in attention and learning [20]. The BG receives inputs from all cortical regions and the thalamus, and efferent pathways project, mostly through BG-thalamo-cortical loops back to the BG [20-22]. Here we report on novel findings in the BG that were measured in HF vs. LF migraineurs.

### 3.1. Basal Ganglia Functional State is Altered in HF Migraineurs

As noted in the results, three salient observations were noted in response to noxious heat. First, with the exception of the NAc, other BG regions showed less activation in HF vs. LF patients. Second, bilateral activation was observed in all regions except the NAc. Third, and perhaps most importantly, single trial averages showed a clear loss of the second peak of the BOLD response to pain.

The observations of decreased activation in HF vs. LF patients in response to noxious heat have not been reported before, although activation in the BG in response to pain had been reported [23]. In some of the BG regions, for example the putamen, acute pain activates the putamen somatotopically [24] an issue which we did not resolve here based on our study design. The relative differences between HF and LF groups observed here is the result of the abnormal late phase BOLD responses. A number of prior studies have shown a biphasic response to noxious heat [25]. While the underlying basis for the second phase is unclear, explanations such as differences in fiber conduction speeds (C fibers vs. A-d fibers) would not explain the differences observed here. Repeated activation of the somatosensory system during migraine have not been shown to affect fiber subtype in the trigeminal system. An alternative explanation might be what we had previously reported on how the changes may reflect alterations in sensory and emotional circuits [25]. Our current data would favor this latter explanation, since unpleasantness ratings, but not intensity ratings differed between the two groups. As noted below, the BG are perhaps ideally located to be involved in integration and response to a noxious stimulus. Our prior brain imaging studies of migraine patients have reported alterations in the BG notably the putamen, with decreased activation in migraine patients vs. controls [17]. The data support the notion that overall the BG function is altered compared with healthy volunteers, and that frequency of attacks would seem to further alter this processing. In addition, standard MR studies of migraine patients have reported lesions [18,19] in the basal ganglia; potentially favoring the interpretation that such changes are caused by the increased migraine frequency rather than causing the frequency to increase. In support of this, migraine is reportedly more frequent in patients with known basal ganglia disorders [26]^,^.

Most imaging studies of acute pain (and other aversive events [27] report decreased activation in the nucleus accumbens in both humans [25,28] and rats [29]. The accumbens is involved in reward processing [27] and increased BOLD activation (here LF > HF) may relate to a relative emotional salience of migraine attacks over time. The increased frequency of migraine attacks in the HF group is thus associated with a decreased hedonic state [30] compared with the LF group.

### 3.2. Alterations in Functional Connectivity (Fc) suggest diffuse alterations in Brain Function

If the basal ganglia play an important role in migraine pathophysiology, altered functional connectivity between different nuclei and other brain regions would be expected. The BG project to or receive inputs from numerous regions including the cingulate, dorsolateral prefrontal cortex (DLPFC), hippocampus and amygdala and such connections have been reported in human imaging studies using diffusion tensor imaging [31]. In our study, we observed Fc differences for all BG subnuclei (used as seeds, see methods) between the two groups. In the case of the putamen and pallidum, increased connectivity in the HF group was observed across some common structures including the anterior insula, the temporal pole and hippocampus. Some of these areas are involved in integrative pain processing such as the anterior insula, the temporal pole and hippocampus. Prior studies from our group have reported alterations in the temporal pole in migraine[17]. The increased Fc associated with the anterior insula may reflect complex processing such as interoceptive processing [32] or integration of pain salience into perceptual decisions [33]. In contrast, decreased connectivity for the caudate was seen for HF < LF migraineurs with temporal pole, anterior insula in addition to the pallidum and middle frontal (MF) cortex. The lower caudate to pallidum Fc in the HF group may be consistent with the known intra BG loops and the low Fc parallels the lower functional activation in the HF group. For the accumbens, an area that stands out for Fc differences is the posterior cingulate cortex. This region is implicated in chronic pain conditions and considered to be important in consciousness and self-reflection [34].

### 3.3. Increased Volume in the Caudate Nucleus in HF Migraineurs

Alterations in function may result in or be produced by alterations in structure. We propose that during migraine attacks, sensory inputs to the basal ganglia via direct (nociceptive pathways) or indirect (thalamo-cortical-basal ganglia loops) pathways [23], are potentially associated with structural changes observed here. Significant inputs from cortical regions including the somatosensory, hippoocampal, orbitofrontal, cindulate and parietal cortex are well described [20,31]. As such these inputs may act as drivers to produce significant increase of volume in the caudate in the HF group. The basis for such changes remains unknown. However, mechanisms that may explain increases in caudate volume include: (i) inflammation [35]; (ii) increased iron accumulation [36]; or (iii) increased dendritic complexity [37]. Most intriguing however, is the paradoxical lower response in caudate to noxious heat in the face of larger volume in the HF group. Increased gray matter volume in the caudate has been described in other brain diseases (e.g., Bipolar Disorder [38], and Schizophrenia [39]), but it should be noted that these increases are opposite to that determined in population studies of caudate volume in healthy subjects where the volume decreases with age [40]. The process may be dynamic, since alterations in caudate volume should be considered as a possible continuum of alterations in the migraine state. It may be a marker for progression, and future studies will be needed to verify that.

### 3.4. Other Considerations

#### Altered Cortico-Thalamic Inputs May Contribute to Altered BG function in Migraine

The basal ganglia receive inputs from many brain regions including the cortex, hippocampus, amygdala and thalamus [41]. It is also the main recipient of dopamine in the brain [42]. Migraine produces hyperexcitable cortical [43,44], and subcortical [45] regions. Given the known connections with these cortical and subcortical regions (involved in symptoms of photophobia, phonophobia, osmophobia and allodynia) and the basal ganglia [20,46] including sensory information [47], these inputs may contribute to alterations observed with increased migraine frequency. Exacerbation of these may be observed as a result of altered chemical integrity within the BG, for example, cortical stimulation in dopamine depleted rats results in abnormal function in basal ganglia circuits [48]. With respect to the latter, altered dopaminergic function [49] and thus alterations in reward function may be present in migraine that may be diminished with increased migraine frequency. The higher response to heat in the HF group in the nucleus accumbens may reflect an alteration in reward systems that have been reported in other chronic pain conditions [50].

As noted above, migraine is associated with increased cortical excitability that has been observed in both in children and adults [51] presumably as a result of increased excitatory systems or decreased inhibitory systems. One potential mechanism of increased caudate volume may thus relate to this altered state in migraine. Indirect clinical support for this comes from a number of studies: (i) In ADHD the caudate is smaller, possibly as a result of diminished inputs or abnormalities of frontal-striatal circuits [52]. (ii) Caudate volume decreases with age [53,54] presumably due to increased cortico-basal ganglia inputs possibly as a result of enhanced cortical inhibitory systems with age; (iii) Caudate volume loss in diseases where there is cortical dysfunction or loss as in dementia or diffuse brain injury [55]. An alternate possibility may relate to the use of medications (here triptans, amount of usage is much higher in HF patients) as has been observed in conditions such as schizophrenia (see [56] (See Caveats). Such alterations may be considered in terms of thalamo-cortico-basal ganglia circuits (see below) where initial sensory drive inputs onto diverse cortical circuits (which over time have increased excitability states) that in turn project back to the basal ganglia and back to the thalamus [21]. Increased sensitivity in thalamic neurons to pain inputs from trigemionvascular systems during both ictal and interictal states may thus drive a sensitized and reverberating circuit that includes the basal ganglia.

#### Neuronal Populations and Basal Ganglia Circuitry

We have recently reported direct inputs from trigeminovascular neurons in the thalamus to the caudate nucleus suggesting direct effects of migraine on basal ganglia function [57]. Differences in responses between low and high frequency migraineurs may relate to alterations in excitatory and inhibitory neurotransmitters. Repeated excitatory inputs acting through glutamatergic receptors in the BG may provide a basis for increased sensitivity of activation in the basal ganglia to noxious stimuli. Disinhibitory effects through GABA mediated receptors modulate synaptic transmission in the basal ganglia [58,59] and contribute to this although complex interactions are likely [59]. Pain may increase excitatory and decreased inhibitory neurotransmission in other brain regions such as the amygdala [60]. Given that the majority of striatal neurons are GABAergic projection neurons, and that these neurons may modulate striatal output [61], glutamatergic activation may induce striatal plasticity including long-term depression (LTD) or activation (LTA) [62]. Thus, the functional and morphological changes may reflect complex alterations due to neurochemical changes in thalamo-cortical-basal ganglia activity with repeated migraine attacks (and perhaps in the interictal period) (see [63]).

#### Further Support for a Putative Role of BG in migraine

Clinical observations may provide some further insights on an association supporting a possible role of the basal ganglia in migraine [64-69,26].

### 3.5. Caveats

#### 3.5.1. Causal Relationship

The cross-sectional study design we used does not allow us to distinguish between cause and effect and thus, we cannot determine with certainty whether the abnormal brain activity and morphometric changes were causing the increased number of migraine attacks or solely caused by them. A longitudinal study would clarify this issue.

#### 3.5.2. Drug Effects

Aside from migraine frequency, the only other major differentiating feature between the two groups was the higher use of non-steroidal anti-inflammatory drugs (NSAIDS) and Triptans in the high frequency group. NSAIDS are commonly used in migraine [70] and may inhibit central sensitization in migraine patients [71] and may also modulate neuronal activity in the trigeminal nucleus in animal studies of central sensitization [72]. These drugs are well-known anti-inflammatory agents, and have a number of effects on brain function (including analgesia) by acting on neuronal and glial systems. Thus, these drugs may also be a confound in this study; however we are unaware of any study evaluating the effects of NSAID's on brain fMRI responses or brain volume. Direct CNS effects of triptans on the other hand are still a matter of debate [73], although several lines of evidence support the presence of direct CNS effects: (i) Triptans, such as zolmitriptan do cross the blood brain barrier [74] which may vary according to their lipid solubility; (ii) Patients display CNS symptoms related to triptans that can be differentiated from placebo [75]; (iii) 3-H labeled sumatriptan binding studies of human brain show increased binding in a number of regions of the brain including the BG with highest binding in the caudate [76]; and (iv) There are some reports of triptan-induced motor changes (dystonia, akathasia) suggestive of actions of these drugs on the BG [76,77] supporting the post-mortem binding studies. The only differences between the groups that we studied were migraine frequency and concomitant use of triptans (increased in the HF Group). Thus our observations may be a result of direct triptan-mediated effects on these structures [78]. Placebo controlled, longitudinal studies are needed to clarify this issue.

## 4. Conclusions

A few studies have attempted to evaluate specific basal ganglia function in pain [14,79,80]. Our findings report significant alterations in structure and function of the basal ganglia in migraineuers as a result of repeated pain, triptan treatment, or both. As such these changes may reflect alterations that may be indicators of migraine chronification/transformation.

## 5. Methods

### 5.1. Subjects and Study Design

**Figure 5:**
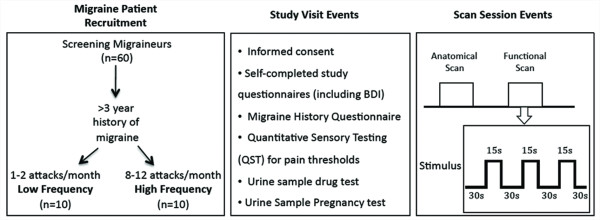
**Subject Recruitment and Experimental Approach**. Based on the frequency of migraine attacks per month, Low frequency (LF) and High frequency (HF) episodic migraine patients were recruited to the study. During the study visit Quantitative Sensory Testing (QST) for pain threshold was performed for each subject and questionnaires were filled out. For each subject morphological (as part of the anatomical scan) and functional (evoked to thermal stimuli) images were acquired as shown in the diagram. Thermal stimuli paradigm is also shown in the diagram.

The study met the criteria of the Helsinki accord for experimentation of pain in human subjects (Helsinki Accord, 1975; http://history.nih.gov/research/downloads/helsinki.pdf) and approved informed consent forms were obtained from all subjects, Figure 5.

Sixty migraine patients were screened for this study out of which, 20 subjects (n = 10 per group) met the inclusion criteria, matched for gender, age, and medication type. The subjects (i) met the criteria for episodic migraine as classified as per the International Classification for Headache (http://www.ihs-classification.org/en/); (ii) had Beck Depression Inventory II (BDI-II) scores ≤ 25; (iii) suffered from episodic migraine for three years or longer; (iv) had no migraine 72 hours prior to the scan and no symptoms of developing one during or 24 hours after the scan; and (v) LF sufferers had 1-2 and HF sufferers had 8-14 headache days per month; and (vi) stable frequency levels were present for at least a year prior to the scan. None of the patients reported the use of either opioids of barbiturates [81]. A detailed history of triptan usage was collected for each subject.

### 5.2. Quantitative Sensory Testing

For all functional studies and for all groups, quantitative sensory testing (QST) was performed using a 1.6 cm × 1.6 cm contact thermode (TSA-II, Medoc Advanced Medical Systems, Ramat Yishai, Israel) prior to the MR session. The temperature increased from a 32°C baseline temperature at the 1^°^C/sec rate until stopped by the subject at the first onset of pain while the corresponding temperature was recorded as the pain threshold (THR).

### 5.3. Noxious Thermal Stimulation

For stimulation during functional imaging, 3 blocks of stimulation (30s baseline/15s stimulation @THR+1) were delivered from a baseline temperature of 32°C using the same probe that was used during QST. The rate of temperature change was 4°C/sec. The 15 seconds pain stimulation period did not include the ramp-up and ramp-down periods of the thermode from the baseline temperature. The ramps were modeled in defining the explanatory variables (EVs) for fMRI data analysis.

### 5.4. Functional and Structural Imaging

All data were collected on a 3 Tesla Siemens Trio scanner with an 8-channel phased array head coil (Erlangen, Germany). For structural data, high resolution, T_1_-weighted datasets were collected from each patient using a 3D MPRAGE pulse sequence (TR/TE/TI = 2100/2.74/1100 ms, FA = 12, 128 sagittal slices, res = 1.33 × 1.0 × 1.0 mm^3^). For acquiring functional data, a Gradient Echo (GE) echo planar imaging (EPI) sequence (TE/TR = 30/2500, res = 3.5 × 3.5 × 3.5 mm^3^, matrix = 64 × 64, 74 volumes, 41 slices) was used.

### 5.5. Data Analysis

#### 5.5.1. Functional Analysis

fMRI analysis was carried out using FMRIB Software Library (FSL) (http://www.fmrib.ox.ac.uk/fsl), version 4.1.3. The initial two volumes were removed from each of the functional scans to allow for signal equilibration. Visual screening of the functional volumes revealed that none of the subjects showed indications of gross movement (> 1 voxel). The skull and other non-brain areas were extracted from the anatomical and functional scans using FSL's script Brain Extraction Tool (BET). Motion Correction using FMRIB's Linear Image Registration Tool (MCFLIRT) was performed on each functional scan. The volumes were spatially smoothed with a 5 mm full-width at half-maximum filter, and a 60s high-pass temporal filter was applied. These functional images were then co-registered with the anatomical images using FMRIB's Linear Image Registration Tool (FLIRT).

First-level fMRI analysis of single subject data was performed using FMRI Expert Analysis Tool (FEAT) Version 5.98. The explanatory variables (EVs) for thermal stimuli were entered using the recorded temperature traces for each subject. Subjects were spatially normalized to the MNI152 brain for group analysis. Group activation maps were generated by fMRI expert analysis tool (FEAT) fMRIB's Local Analysis of Mixed Effects (FLAME). For all of the functional comparisons, the group statistical parametric maps were threshold using a Gaussian Mixture Model (GMM) technique, a multiple comparisons-based analysis generally used for unsupervised classification of data into multiple categories (Pendse et al., 2007; Moulton et al., 2007). Single trial averages (STAs) were calculated using in-house programs.

#### 5.5.2. Structural Analysis

Subcortical volumetric segmentation was performed with the Freesurfer image analysis software (http://surfer.nmr.mgh.harvard.edu/). The initial processing steps included (i) Motion correction and averaging of the two volumetric T_1_-weighted MPRAGE images, (ii) Removal of non-brain tissue using a hybrid watershed/surface deformation procedure [82], (iii) Automated Talairach transformation, (iv) Segmentation of the subcortical white matter and deep gray matter volumetric structures (including hippocampus, amygdala, caudate, putamen, ventricles) [83,84], (v) Intensity normalization [85]. Subsequent to these processing steps, the volumes were labeled based on both subject-independent probabilistic atlas and subject-specific measured values [83,84]. These labels were then mapped into Talairach space to achieve point-to-point correspondence for all subjects. This method uses both intensity and continuity information from the entire 3-dimensional high resolution structural volume in segmentation. A univariate analysis of variance for each of the segmented volumes was performed separately using IBM SPSS 19.0 statistics package to assess the differences between the two groups of migraine patients while accounting for the differences in the cranium size [86] and age as additional regressors.

#### 5.5.3. Functional Connectivity (Fc) Analysis

Functional connectivity was measured using a seed correlation based approach [87,88]. The evoked functional connectivity was assessed in order to determine if there were any differences in the functional connectivity of each of the seeds of interest to the network they functionally connected to for pain processing between the two cohorts. Seeds/Regions chosen for functional connectivity analysis were defined anatomically for the basal ganglia nuclei and also additional subcortical areas (PAG, Pulvinar and Hypothalamus) based on considerations that functional connectivity with those areas may reflect important processing of migraine systems between the two groups based on our previous studies in migraine [17]. The basal ganglia ROIs were defined by automatic segmentation of the T1-weighted anatomical volumes for each subject individually using Freesurfer image analysis software (http://surfer.nmr.mgh.harvard.edu/). Other ROIs were defined on the MNI brain in the standard space and then transformed to each subject's anatomical space.

Preprocessing steps were similar to the steps described for functional analysis above. For each subject the WM and CSF masks were created in anatomical space using Freesurfer tools (http://surfer.nmr.mgh.harvard.edu/). All time-courses in the brain were orthogonalized with respect to the eigen time-courses of WM and CSF masks which were computed by singular value decomposition (SVD) [89]. fMRI time-courses from each seed ROI were also extracted using SVD. The time courses were normalized for General Linear Model (GLM) analysis. The resulting GLM analysis parameter estimates (correlation coefficients) were transformed into normally distributed quantities using a Fisher z-transform, registered to MNI space and entered into a mixed effects group analysis (FLAME1). The group statistical parametric maps were threshold using a GMM technique (see above).

## Abbreviations

**BDI**: Beck Depression Inventory; **BOLD**: Blood Oxygenation Level Dependent; **CSF**: Cerebrospinal Fluid; **EPI**: Echo Planar Imaging; **Fc**: Functional Connectivity; **FEAT**: fMRI expert analysis tool; **FLAME**: fMRIB's Local Analysis of Mixed Effects; **fMRI**: Functional Magnetic Resonance Imaging; **GE**: Gradient Echo; **GLM**: General Linear Model; **GMM**: Gaussian Mixture Model; **MPRAGE**: Magnetization Prepared Rapid Acquisition Gradient Echo; **NAc**: Nucleus Accumbens; **PAG**: Periaqueductal Gray; **QST**: Quantitative Sensory Testing; **ROI**: Region of Interest; **STA**: Single Trial Average; **SVD**: singular value decomposition; **THR**: Pain Threshold; **WM**: White Matter.

## Competing interests

Dr.Bigal is a full time employee of Merck Inc. He owns stocks and stock options. Other authors have no competing interest to declare.

## Authors' contributions

NM carried out experiments, carried out analysis, wrote first draft of paper; LB helped with analysis; GP carried out statistical analysis; LN & JB recruited patients, collected data, carried out experiments; MB & RB helped write the paper; DB conceptualized study, designed the experiments and helped write the paper. All authors read and approved the final manuscript.
